# Peripheral blood non-canonical small non-coding RNAs as novel biomarkers in lung cancer

**DOI:** 10.1186/s12943-020-01280-9

**Published:** 2020-11-12

**Authors:** Wanjun Gu, Junchao Shi, Hui Liu, Xudong Zhang, Jin J. Zhou, Musheng Li, Dandan Zhou, Rui Li, Jingzhu Lv, Guoxia Wen, Shanshan Zhu, Ting Qi, Wei Li, Xiaojing Wang, Zhaohua Wang, Hua Zhu, Changcheng Zhou, Kenneth S. Knox, Ting Wang, Qi Chen, Zhongqing Qian, Tong Zhou

**Affiliations:** 1 State Key Laboratory of Bioelectronics, School of Biological Sciences and Medical Engineering, Southeast University, 2 Sipailou, Nanjing, 210096 Jiangsu China; 2 Division of Biomedical Sciences, School of Medicine, University of California, Riverside, 900 University Avenue, Riverside, CA 92521 USA; 3 Anhui Province Key Laboratory of Immunology in Chronic Diseases, Anhui Key Laboratory of Infection and Immunity, Department of Laboratory Medicine, Bengbu Medical College, 2600 Donghaidadao, Bengbu, 233003 Anhui China; 4 Department of Epidemiology and Biostatistics, University of Arizona, Tucson, AZ 85721 USA; 5 Department of Physiology and Cell Biology, University of Nevada, Reno School of Medicine, 1664 North Virginia Street, Reno, Nevada, 89557 USA; 6 Anhui Clinical and Preclinical Key Laboratory of Respiratory Disease, Department of Respiration, First Affiliated Hospital, Bengbu Medical College, Bengbu, 233000 Anhui China; 7 The Infectious Disease Hospital of Bengbu City, Bengbu, 233000 Anhui China; 8 Department of Surgery, The Ohio State University, Columbus, OH 43210 USA; 9 Department of Internal Medicine, College of Medicine Phoenix, University of Arizona, Phoenix, AZ 85004 USA

**Keywords:** Lung cancer, Tuberculosis, tsRNA, rsRNA, ysRNA

## Abstract

One unmet challenge in lung cancer diagnosis is to accurately differentiate lung cancer from other lung diseases with similar clinical symptoms and radiological features, such as pulmonary tuberculosis (TB). To identify reliable biomarkers for lung cancer screening, we leverage the recently discovered non-canonical small non-coding RNAs (i.e., tRNA-derived small RNAs [tsRNAs], rRNA-derived small RNAs [rsRNAs], and YRNA-derived small RNAs [ysRNAs]) in human peripheral blood mononuclear cells and develop a molecular signature composed of distinct ts/rs/ysRNAs (TRY-RNA). Our TRY-RNA signature precisely discriminates between control, lung cancer, and pulmonary TB subjects in both the discovery and validation cohorts and outperforms microRNA-based biomarkers, which bears the diagnostic potential for lung cancer screening.

**Supplementary Information:**

The online version contains supplementary material available at 10.1186/s12943-020-01280-9.

One unmet challenge in current lung cancer diagnosis is to accurately differentiate lung cancer from other lung diseases with similar clinical symptoms and radiological features. Imaging-based screening methods, such as low-dose computed topography (LDCT), could sometimes be false positives, as indeterminate pulmonary nodules may also be caused by other lung diseases such as pulmonary tuberculosis (TB) [1], which is especially concerning for clinical practice in TB-endemic countries/regions. Therefore, additional noninvasive diagnostic procedures are much-needed to avoid a misdiagnosis in patients with lung cancer mimicking pulmonary TB, or vice versa. Here, we aim to develop a peripheral blood mononuclear cell (PBMC)-based molecular signature to differentiate lung cancer patients from healthy controls and pulmonary TB patients by harnessing the novel small non-coding RNAs (sncRNAs).

Recent sncRNA sequencing (sncRNA-seq) attempts have ubiquitously detected several non-canonical sncRNA types, which are fragments derived from canonically transcribed parent large RNAs, including tRNA-derived small RNAs (tsRNAs), rRNA-derived small RNAs (rsRNAs), and YRNA-derived small RNAs (ysRNAs) [2]. ts/rs/ysRNAs have been discovered in a wide range of species [2]. The biological functions of tsRNAs have become a recent highlight and been linked with various human diseases [3], including cancers [4–6], while rsRNAs and ysRNAs show sensitive response to pathophysiological conditions [6, 7]. In this study, we develop a diagnostic signature composed of distinct ts/rs/ysRNAs (TRY-RNA) in human PBMCs. Our TRY-RNA signature accurately discriminates between control, lung cancer, and pulmonary TB subjects in both the discovery and validation cohorts and outperforms microRNA (miRNA)-based biomarkers. Fig. S1 (Additional file 1) provides an overview of the experimental design.

## Dysregulated non-canonical sncRNAs in lung cancer

**Fig. 1:**
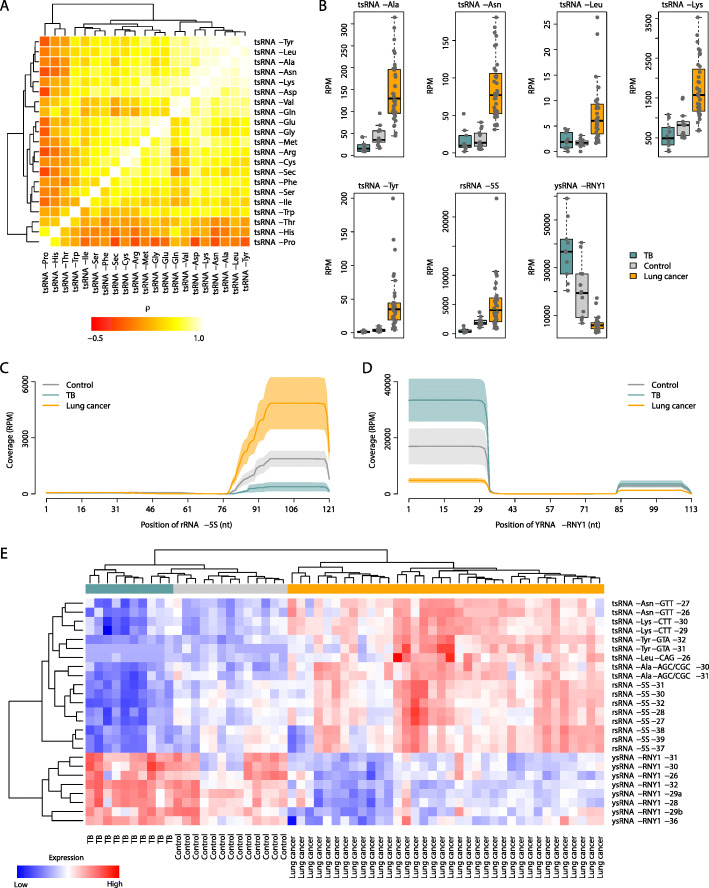
The dysregulated non-canonical sncRNAs in lung cancer. **a** The co-expression pattern tsRNA subcategories across the PBMC samples in the discovery cohort. The correlation coefficient was calculated by *Spearman*’s rank correlation test. tsRNA-Ala, tsRNA-Asn, tsRNA-Leu, tsRNA-Lys, and tsRNA-Tyr were strongly and positively correlated with each other. **b** The expression profile of tsRNA-Ala, tsRNA-Asn, tsRNA-Leu, tsRNA-Lys, tsRNA-Tyr, rsRNA-5S, and ysRNA-RNY1 among the control, lung cancer, and TB subjects. *RPM*: reads per million. **c** and **d** The coverage profile of the PBMC rsRNA-5S and ysRNA-RNY1 sequences along rRNA-5S and YRNA-RNY1, respectively. The solid curves indicate the mean *RPM* values for the control, lung cancer, and TB groups. The colored bands represent the 95% confidence interval. nt: nucleotide. rsRNA-5S sequences were primarily derived from the 3′-end of rRNA-5S, whereas ysRNA-RNY1 sequences were largely derived from the 5′-end of YRNA-RNY1 with a small portion of fragments mapped to the 3′-end of YRNA-RNY1. **e** Expression heatmap of the sncRNA species within the TRY-RNA signature in the discovery cohort

We performed sncRNA-seq for the PBMC samples collected from 59 human subjects in the discovery cohort, including 13 healthy controls, 10 pulmonary TB patients, and 36 lung cancer patients (Additional file 2: Table S1). The raw sequencing data were processed by our newly developed computational framework, *SPORTS1.0*, which was designed to optimize the annotation and quantification of non-canonical sncRNAs (i.e.*,* tsRNAs, rsRNAs, and ysRNAs) in addition to miRNAs [2]. In total, 6673 tsRNA species, 20,172 rsRNA species, 1238 ysRNA species, and 973 miRNA species were identified in human PBMCs (Additional file 1: Fig. S2). We investigated the co-expression pattern of tsRNAs across the PBMC samples in the discovery cohort by grouping tsRNA species into subcategories according to their parent tRNA types. We found that the expression of the tsRNAs derived from the tRNAs of alanine (tsRNA-Ala), asparagine (tsRNA-Asn), leucine (tsRNA-Leu), lysine (tsRNA-Lys), and tyrosine (tsRNA-Tyr) was strongly and positively correlated with that of each other (*Spearman*’s rank correlation test: *ρ* > 0.700 and *P* < 10^− 9^) (Fig. 1a), suggesting shared biogenesis pathways among these tsRNAs. Interestingly, tsRNA-Ala, tsRNA-Asn, tsRNA-Leu, tsRNA-Lys, and tsRNA-Tyr were the only five tsRNA groups that were upregulated in the lung cancer patients relative to the controls (adjusted *P* < 0.05) (Fig. 1b). We further found that the expression of these five tsRNA groups was also significantly higher in the lung cancer patients than in the pulmonary TB subjects (*P* < 0.05) (Fig. 1b). We next grouped rsRNA and ysRNA species into subcategories according to their parent rRNA/YRNA types and found that the rsRNAs derived from rRNA-5S (rsRNA-5S) were significantly upregulated in the lung cancer patients relative to the controls, while the ysRNAs originating from YRNA-RNY1 (ysRNA-RNY1) were downregulated in the lung cancer patients compared with the controls (adjusted *P* < 0.05) (Fig. 1b). More interestingly, the expression of rsRNA-5S and ysRNA-RNY1 showed a completely inverse pattern in the pulmonary TB patients: rsRNA-5S was significantly downregulated in the TB patients relative to the controls, while ysRNA-RNY1 was upregulated in the TB patients compared with the controls (*P* < 0.05) (Fig. 1b). We further mapped the individual tsRNA-Ala, tsRNA-Asn, tsRNA-Leu, tsRNA-Lys, tsRNA-Tyr, rsRNA-5S, and ysRNA-RNY1 species to the corresponding parent RNAs and identified a nonrandom fragmentation pattern (Fig. 1c-d and Additional file 1: Fig. S3), suggesting highly regulated biogenesis of these sncRNAs. In addition, we investigated the association of these non-canonical sncRNA expression with cancer stage, histological type, lymph node status, metastasis status, and smoking history, but no significant difference was observed (Additional file 1: Fig. S4-S8).

## The molecular signature composed of non-canonical sncRNAs

We next develop a molecular signature of sncRNAs by harnessing the above prioritized sncRNA subcategories (i.e.*,* tsRNA-Ala, tsRNA-Asn, tsRNA-Leu, tsRNA-Lys, tsRNA-Tyr, rsRNA-5S, and ysRNA-RNY1). In total, nine tsRNA species, eight rsRNA species, and eight ysRNA species (Additional file 1: Fig. S9A-C) were selected, which consisted of a molecular signature with 25 distinct non-canonical sncRNAs (Additional file 1: Fig. S9D and Additional file 2: Table S2), referred to as the TS/RS/YS-RNA (TRY-RNA) signature. Both principal component analysis (Additional file 1: Fig. S9E) and hierarchical clustering on RNA expression (Fig. 1e) confirmed the discriminative power of the TRY-RNA signature between the control, lung cancer, and TB groups in the discovery cohort. To systematically evaluate the classification power of the TRY-RNA signature, a TRY-RNA index was assigned to each subject based on the expression of the ts/rs/ysRNAs within the TRY-RNA signature (Additional file 3: Methods). The TRY-RNA index was a linear combination of the expression values of the sncRNA species within the TRY-RNA signature. A higher TRY-RNA index implies a higher likelihood of lung cancer. We found that the TRY-RNA index was significantly higher in the lung cancer patients than in the healthy controls, while the TRY-RNA index of the pulmonary TB patients was significantly lower than that of the controls (*t*-test: *P* < 10^− 5^) (Additional file 1: Fig. S10A). The area under the receiver operating characteristic (ROC) curve (*AUC*) was 1.000 between the cancer and non-cancer subjects and 0.994 between the TB and non-TB subjects (Additional file 1: Fig. S10B). In addition, we investigated the association of the expression of the individual RNA species within the TRY-RNA signature with cancer stage, histological type, lymph node status, metastasis status, and smoking history, but significant difference was only observed for ysRNA-RNY1–28 and ysRNA-RNY1-29a between adenocarcinoma and squamous cell carcinoma patients (Additional file 1: Fig. S11-S15).

## The performance of the TRY-RNA signature in the validation cohort

**Fig. 2:**
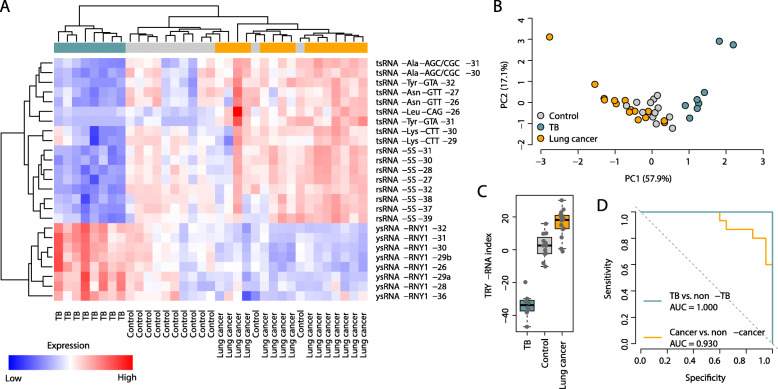
The performance of the TRY-RNA signature in the validation cohort. **a** Expression heatmap of the sncRNA species within the TRY-RNA signature in the validation cohort. **b** Principal component analysis of the TRY-RNA signature. PC1: the first principal component; PC2: the second principal component. PC1 significantly differed between the controls and lung cancer patients (*t*-test: *P* = 3.1 × 10^− 3^), between the controls and TB patients (*t*-test: *P* = 4.7 × 10^− 6^), and between the lung cancer and TB patients (*t*-test: *P* = 4.1 × 10^− 8^). **c** Comparison of the TRY-RNA index among the control, lung cancer, and TB subjects in the validation cohort. **d** The ROC curve of the TRY-RNA index in distinguishing between lung cancer and non-cancer subjects and between TB and non-TB subjects in the validation cohort

We further assessed the TRY-RNA signature in the validation cohort with 35 human PBMC samples collected from 12 healthy controls, 15 lung cancer patients, and 8 pulmonary TB patients (Additional file 2: Table S3). Unsupervised hierarchical clustering and principal component analysis demonstrated a totally distinct expression pattern of the TRY-RNA signature between the lung cancer and TB subjects, with the controls largely falling in between in the validation cohort (Fig. 2a-b). The TRY-RNA index in the validation cohort was significantly higher in the lung cancer patients than in the healthy controls, while the TRY-RNA index of the TB patients was significantly lower than that of the controls (*t*-test: *P* < 0.005) (Fig. 2c). The *AUC* was 0.930 between the cancer and non-cancer subjects and 1.000 between the TB and non-TB subjects (Fig. 2d), which suggests the strong classification power of the TRY-RNA signature for both lung cancer and pulmonary TB screening.

## Comparison between the TRY-RNA and miRNA-based signatures

We also compared the expression profile of miRNAs among the control, lung cancer, and pulmonary TB subjects in the discovery cohort and identified a signature with 43 miRNA species, referred to as the MIR signature (Additional file 1: Fig. S16 and Additional file 2: Table S4). Similar to the TRY-RNA signature, we assigned a MIR index to each subject based on the expression of the miRNAs within the MIR signature. We found that in both the discovery and validation cohorts, while the MIR index can differentiate the lung cancer patients from the control and TB subjects (Additional file 1: Fig. S17 and S18A-B), a resampling test (Additional file 3: Methods) demonstrated a superior classification power of the TRY-RNA signature compared to the MIR signature (Additional file 1: Fig. S18C). We further investigated whether the MIR signature provided additive classification power to the TRY-RNA signature by combining both signatures (referred to as the TRY-RNA∪MIR signature). Although the performance of the TRY-RNA∪MIR signature was fairly good, the TRY-RNA∪MIR signature didn’t outperform the TRY-RNA signature and barely provided additive information for the classification (Additional file 1: Fig. S18D-F).

## Conclusions

Our TRY-RNA signature derived from the repertoire of PBMC non-canonical sncRNAs makes it possible for the early diagnosis of lung cancer and pulmonary TB, which may reflect the host responses to different antigens and would represent an improvement over the previous studies focusing solely on tsRNAs in cancer tissues [4, 5, 8]. Interestingly, the performance of the TRY-RNA signature shows superiority over the miRNA-based signature, which could be due to the more complex layer of non-canonical sncRNAs. For example, tsRNAs and rsRNAs exhibit an unexpected complexity in regards to their RNA modifications as well as their sequence diversities [9]. Our previous study suggests that both tsRNAs and rsRNAs are involved in mammalian epigenetic inheritance, which form a “RNA code” to convey environmental clue to the offspring [7, 10]. Also, tsRNAs are thought to regulate translation process and ribosome biogenesis in versatile ways, including the fine-tuning of the ribosome composition that may affect the translational specificity on a selective pool of mRNAs (also referred to as ribosome heterogeneity). In other words, change in tsRNA (and perhaps rsRNA/ysRNA as well) composition may result in altered ribosome heterogeneity that directs the cell to a specific functional state [9]. The complexity and possible permutations of different tsRNA/rsRNA/ysRNAs may endow the superior information capacity and specificity that are needed to distinguish complex diseases, and as being harnessed here, represent a “disease RNA code” in lung cancer screening.

## Supplementary Information

**Additional file 1: **Supplementary Figures. **Fig. S1.** The workflow of the study. **Fig. S2.** The landscape of non-canonical sncRNAs in human PBMCs. **Fig. S3.** The mapping profile of tsRNAs. **Fig. S4.** Comparison of the expression of the prioritized sncRNA subcategories between lung cancer stages. **Fig. S5.** Comparison of the expression of the prioritized sncRNA subcategories between lung cancer histological types. **Fig. S6.** Comparison of the expression of the prioritized sncRNA subcategories between the lung cancer patients with and without lymph node involvement. **Fig. S7.** Comparison of the expression of the prioritized sncRNA subcategories between the lung cancer patients with and without distant metastasis. **Fig. S8.** Comparison of the expression of the prioritized sncRNA subcategories between the lung cancer patients with and without smoking history. **Fig. S9.** The TRY-RNA signature. **Fig. S10.** The TRY-RNA index in the discovery cohort. **Fig. S11.** Comparison of the expression of the sncRNA species within the TRY-RNA signature between lung cancer stages. **Fig. S12.** Comparison of the expression of the sncRNA species within the TRY-RNA signature between lung cancer histological types. **Fig. S13.** Comparison of the expression of the sncRNA species within the TRY-RNA signature between the lung cancer patients with and without lymph node involvement. **Fig. S14.** Comparison of the expression of the sncRNA species within the TRY-RNA signature between the lung cancer patients with and without distant metastasis. **Fig. S15.** Comparison of the expression of the sncRNA species within the TRY-RNA signature between the lung cancer patients with and without smoking history. **Fig. S16.** Expression heatmap of the MIR signature in the discovery cohort. **Fig. S17.** The MIR index in the discovery cohort. **Fig. S18.** Comparison between the TRY-RNA and MIR signatures.

**Additional file 2: **Supplementary Tables. **Table S1.** The human subjects of the discovery cohort. **Table S2.** The TRY-RNA signature. **Table S3.** The human subjects of the validation cohort. **Table S4.** The MIR signature.

**Additional file 3.** Methods.

## Data Availability

The datasets generated and analyzed during the current study are available in the Gene Expression Omnibus (https://www.ncbi.nlm.nih.gov/geo/) under the accession number GSE148861 and GSE148862 for the discovery and validation cohorts, respectively.

## Abbreviations

LDCT: Low-dose computed topography
TB: Tuberculosis
miRNA: MicroRNA
PBMC: Peripheral blood mononuclear cell
EV: Extracellular vesicle
sncRNA: Small non-coding RNA
sncRNA-seq: Small non-coding RNA sequencing
tsRNA: tRNA-derived small RNA
rsRNA: rRNA-derived small RNAs
ysRNA: YRNA-derived small RNAs
nt: Nucleotides
*RPM*: Reads per million
ROC: Receiver operating characteristic
*AUC*: Area under the receiver operating characteristic curve
