# Supplementary material for: Peripheral blood non-canonical small non-coding RNAs as novel biomarkers in lung cancer
Source: Mol Cancer. 2020 Nov 12;19:159. doi: 10.1186/s12943-020-01280-9 (PMC7659116; doi:10.1186/s12943-020-01280-9)
Supplement: Supplementary file 1 — Additional file 1: Supplementary Figures. Fig. S1. The workflow of the study. Fig. S2. The landscape of non-canonical sncRNAs in human PBMCs. Fig. S3. The mapping profile of tsRNAs. Fig. S4. Comparison of the expression of the prioritized sncRNA subcategories between lung cancer stages. Fig. S5. Comparison of the expression of the prioritized sncRNA subcategories between lung cancer histological types. Fig. S6. Comparison of the expression of the prioritized sncRNA subcategories between the lung cancer patients with and without lymph node involvement. Fig. S7. Comparison of the expression of the prioritized sncRNA subcategories between the lung cancer patients with and without distant metastasis. Fig. S8. Comparison of the expression of the prioritized sncRNA subcategories between the lung cancer patients with and without smoking history. Fig. S9. The TRY-RNA signature. Fig. S10. The TRY-RNA index in the discovery cohort. Fig. S11. Comparison of the expression of the sncRNA species within the TRY-RNA signature between lung cancer stages. Fig. S12. Comparison of the expression of the sncRNA species within the TRY-RNA signature between lung cancer histological types. Fig. S13. Comparison of the expression of the sncRNA species within the TRY-RNA signature between the lung cancer patients with and without lymph node involvement. Fig. S14. Comparison of the expression of the sncRNA species within the TRY-RNA signature between the lung cancer patients with and without distant metastasis. Fig. S15. Comparison of the expression of the sncRNA species within the TRY-RNA signature between the lung cancer patients with and without smoking history. Fig. S16. Expression heatmap of the MIR signature in the discovery cohort. Fig. S17. The MIR index in the discovery cohort. Fig. S18. Comparison between the TRY-RNA and MIR signatures. [file 12943_2020_1280_MOESM1_ESM.pdf]

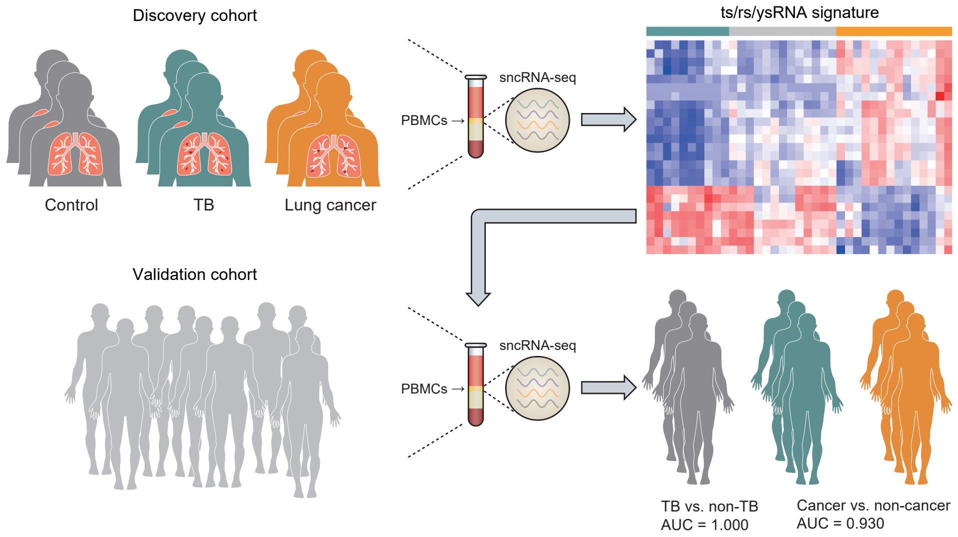

Figure S1. The workflow of the study. PBMC ts/rs/ysRNA expression of the human subjects in the discovery cohort was profiled by sncRNA-seq. A molecular signature composed of ts/rs/ysRNAs was developed to discriminate between healthy controls, lung cancer patients, and pulmonary TB subjects. This signature was validated in the validation cohort with high accuracy. *AUC*: area under the receiver operating characteristic curve.

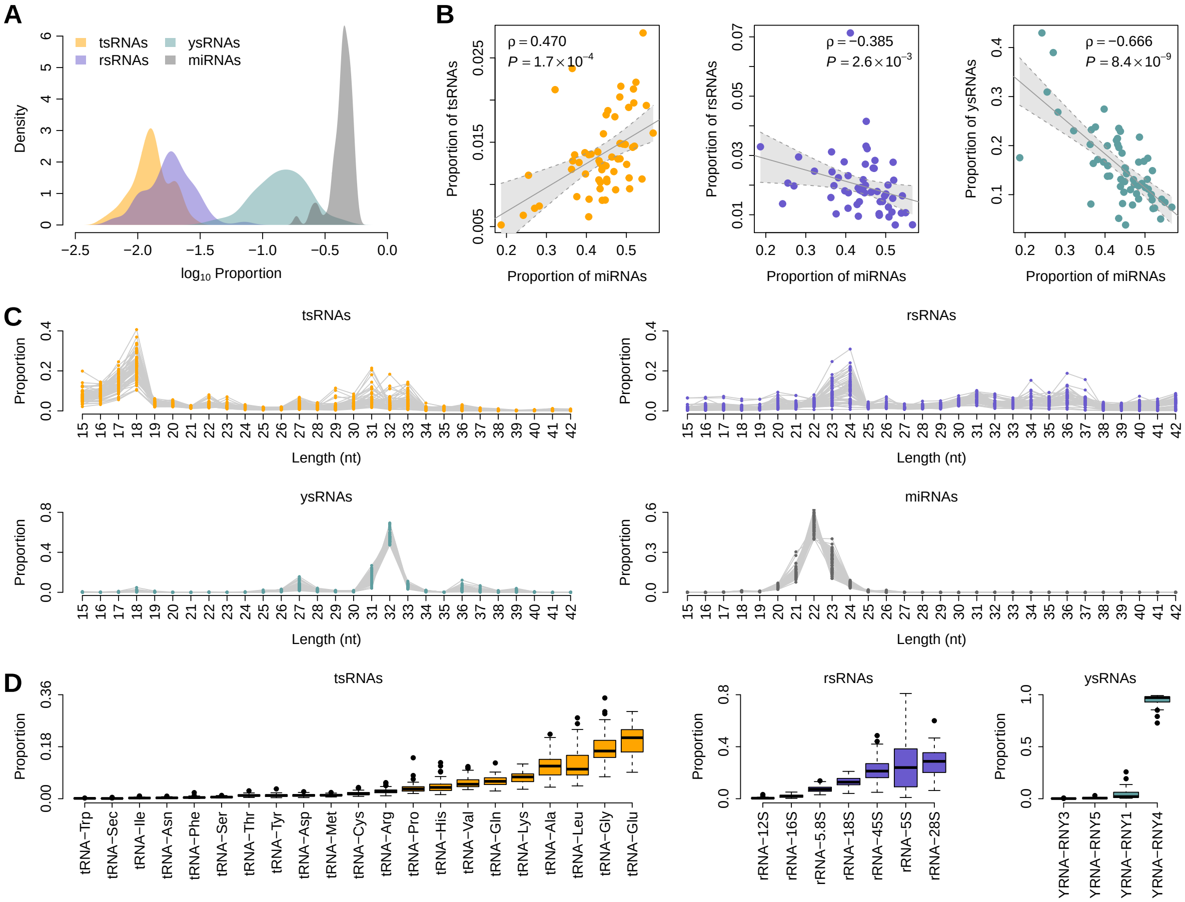

Figure S2. The landscape of non-canonical sncRNAs in human PBMCs. (A) The distribution of sncRNA read proportions. The X-axis was $\log_{10}$-transformed. For each sample, we computed the read proportions of tsRNAs, rsRNAs, ysRNAs, and miRNAs, respectively. Using the current standard sncRNA-seq library construction protocol, the read proportion of miRNAs was significantly higher than that of tsRNAs, rsRNAs, and ysRNAs (*Wilcoxon* signed-rank test: $P < 10^{-10}$). (B) The correlation in read proportions between miRNAs and non-canonical sncRNAs, *i.e.*, tsRNAs, rsRNAs, and ysRNAs. The correlation coefficients (ρ) and P-values were calculated by *Spearman*'s rank correlation test. A positive correlation in read proportion was observed between tsRNAs and miRNAs across all PBMC samples, whereas the read proportion of rsRNAs and ysRNAs was negatively correlated with that of miRNAs. (C) The length distribution of sncRNAs. Each dot represents one PBMC sample. The Y-axis shows the read proportion within each sncRNA category, *i.e.*, tsRNA, rsRNA, ysRNA, and miRNA. As expected, miRNAs were generally 20-24 nucleotides (nts) in length. However, the size pattern of tsRNAs, rsRNAs, and ysRNAs was more complicated with multiple distribution peaks, which suggests distinct biogenesis pathways and functional roles for non-canonical sncRNAs relative to miRNAs. (D) The parent large RNAs from which non-canonical sncRNAs originated. The Y-axis shows the read proportion within each non-canonical sncRNA category, *i.e.*, tsRNA, rsRNA, and ysRNA. Most of the PBMC tsRNAs were derived from the tRNA-Ala, tRNA-Leu, tRNA-Gly, and tRNA-Glu; a large proportion of the rsRNAs were derived from 45S, 5S, and 28S rRNAs; over two-thirds of the ysRNAs were derived from YRNA-RNY4, with those derived from YRNA-RNY1 being the second most common.

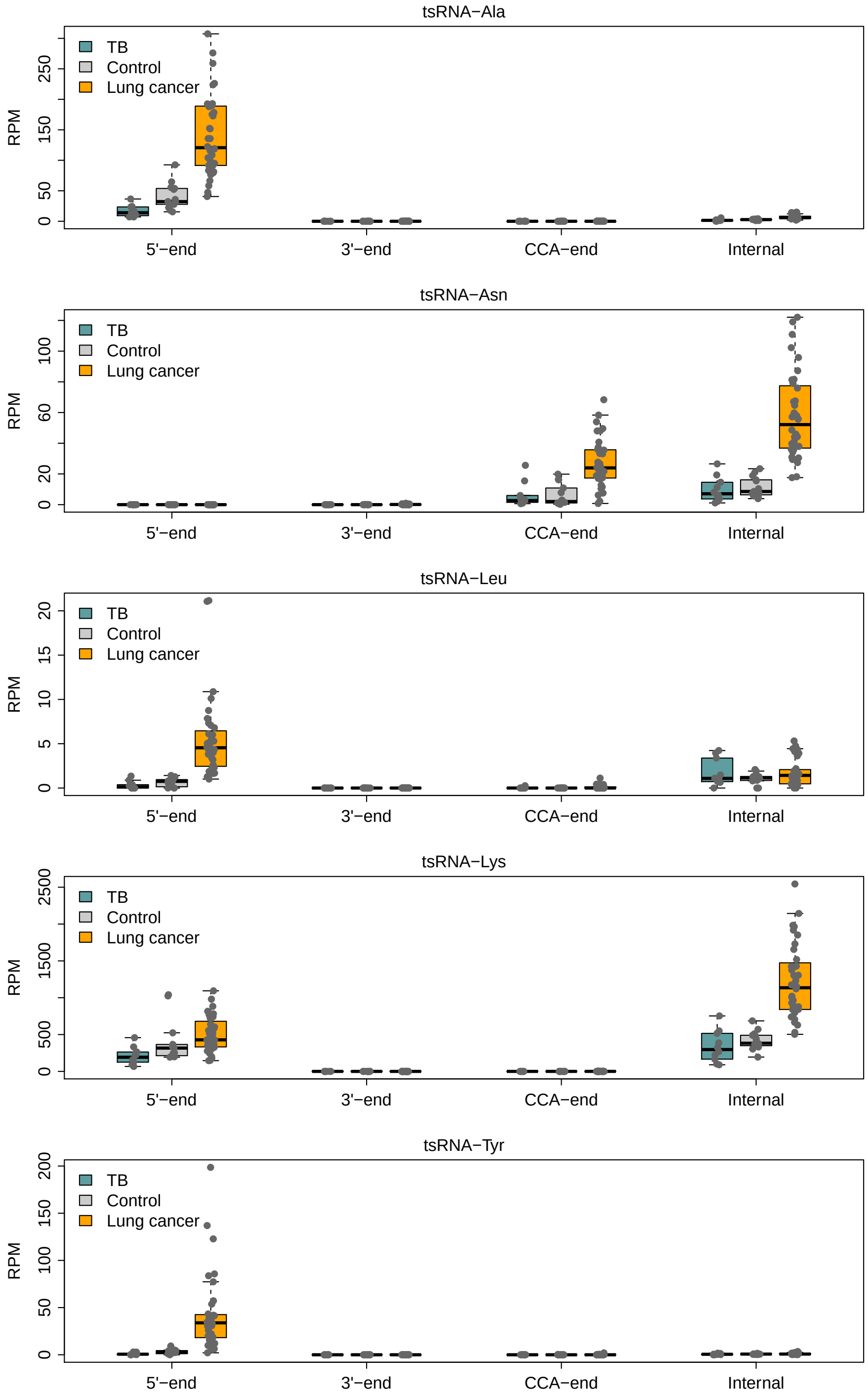

Figure S3. The mapping profile of tsRNAs. We classified individual tsRNAs according to the fragment locations on the corresponding parent tRNAs, *i.e.*, the 5 terminus, 3 terminus, 3 CCA-end, or internal region of tRNAs. tsRNA-Ala and tsRNA-Tyr were primarily mapped to the tRNA 5-end; tsRNA-Leu and tsRNA-Lys were largely mapped to the 5-end and internal regions of tRNAs, whereas tsRNA-Asn sequences were primarily mapped to the CCA-end and internal regions of tRNAs.

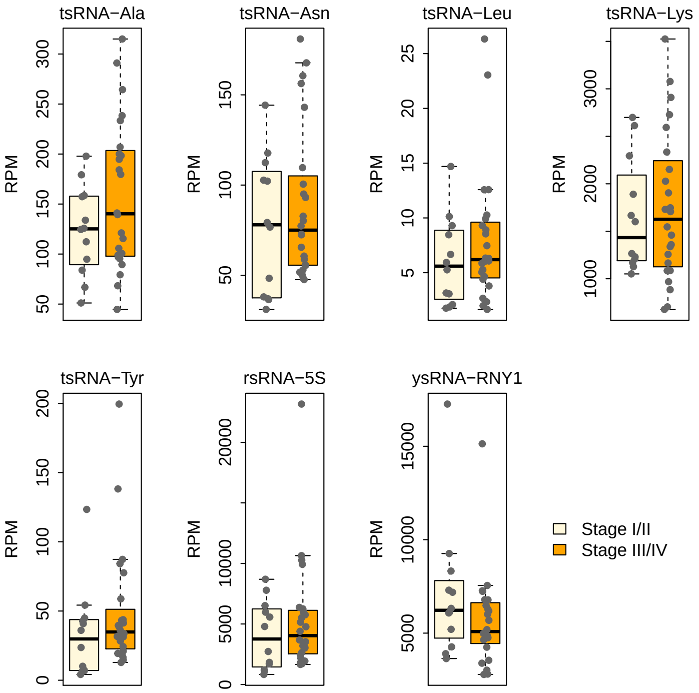

Figure S4. Comparison of the expression of the prioritized sncRNA subcategories between lung cancer stages. Groupwise comparisons (stage I/II *vs.* III/IV) were performed using a linear model controlling for age and sex. No significant difference was observed (adjusted $P > 0.05$).

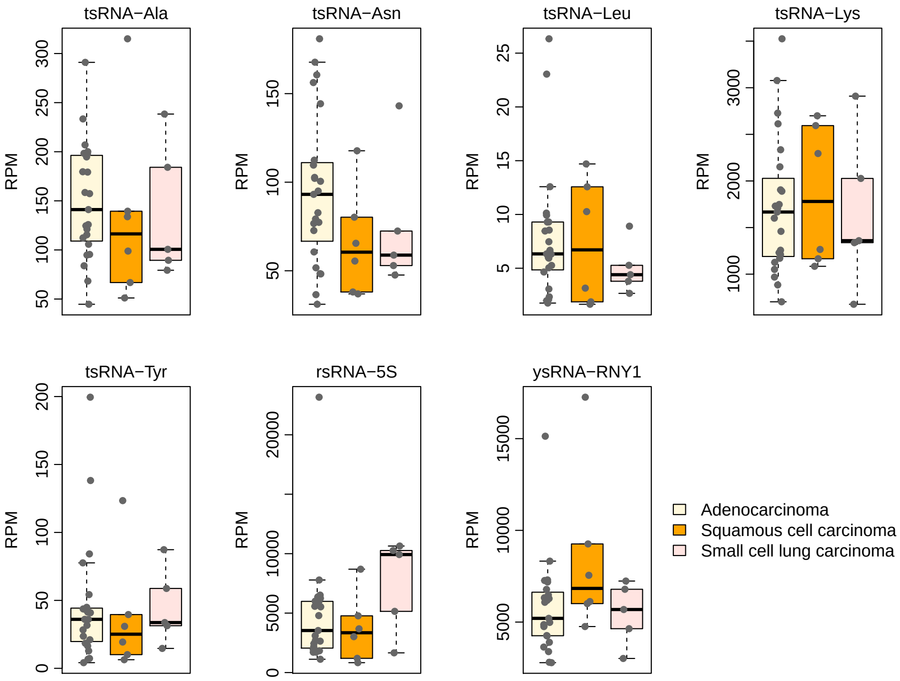

Figure S5. Comparison of the expression of the prioritized sncRNA subcategories between lung cancer histological types. Groupwise comparisons (*i.e.*, adenocarcinoma *vs.* squamous cell carcinoma, adenocarcinoma *vs.* small cell lung carcinoma, and squamous cell carcinoma *vs.* small cell lung carcinoma) were performed using a linear model controlling for age and sex. No significant difference was observed (adjusted $P > 0.05$).

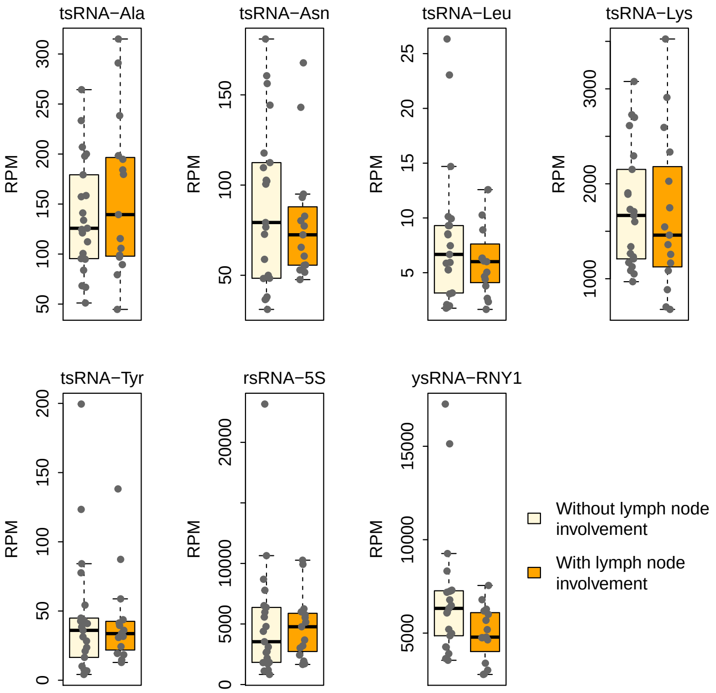

Figure S6. Comparison of the expression of the prioritized sncRNA subcategories between the lung cancer patients with and without lymph node involvement. Groupwise comparisons were performed using a linear model controlling for age and sex. No significant difference was observed (adjusted $P > 0.05$).

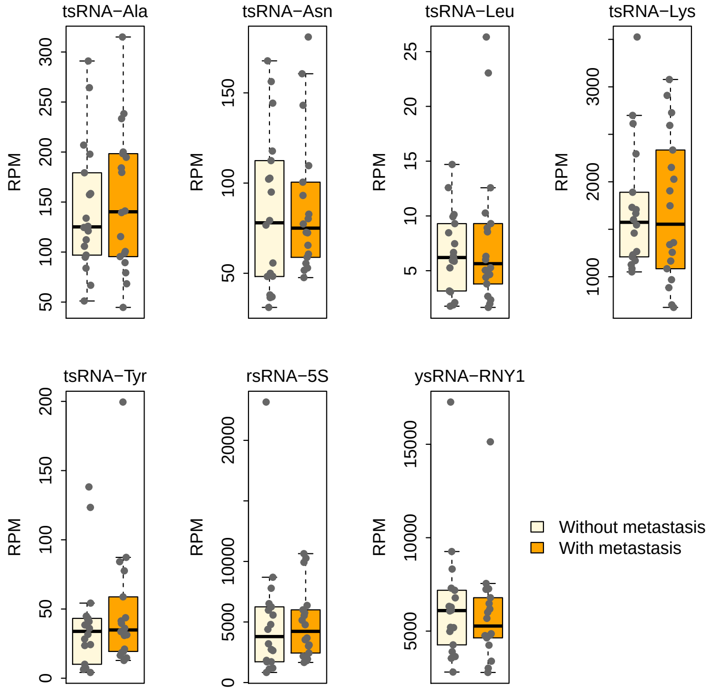

Figure S7. Comparison of the expression of the prioritized sncRNA subcategories between the lung cancer patients with and without distant metastasis. Groupwise comparisons were performed using a linear model controlling for age and sex. No significant difference was observed (adjusted $P > 0.05$).

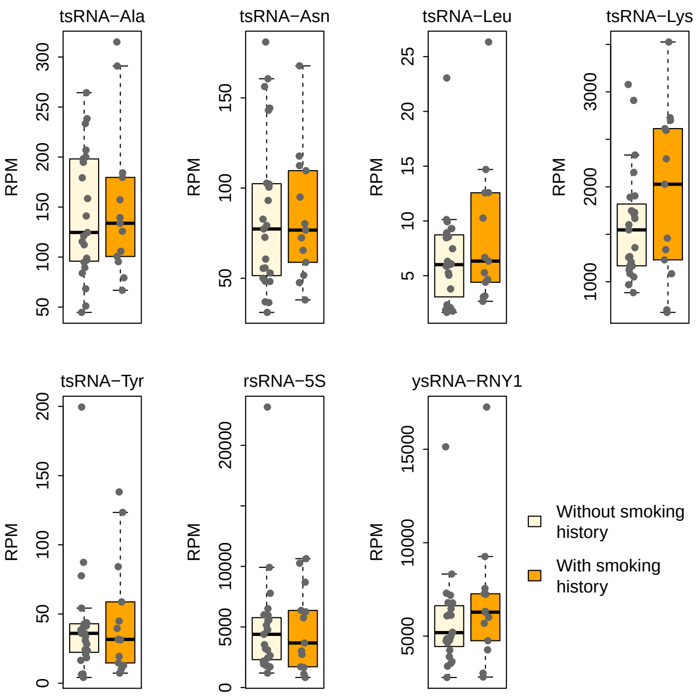

Figure S8. Comparison of the expression of the prioritized sncRNA subcategories between the lung cancer patients with and without smoking history. Groupwise comparisons were performed using a linear model controlling for age and sex. No significant difference was observed (adjusted $P > 0.05$).

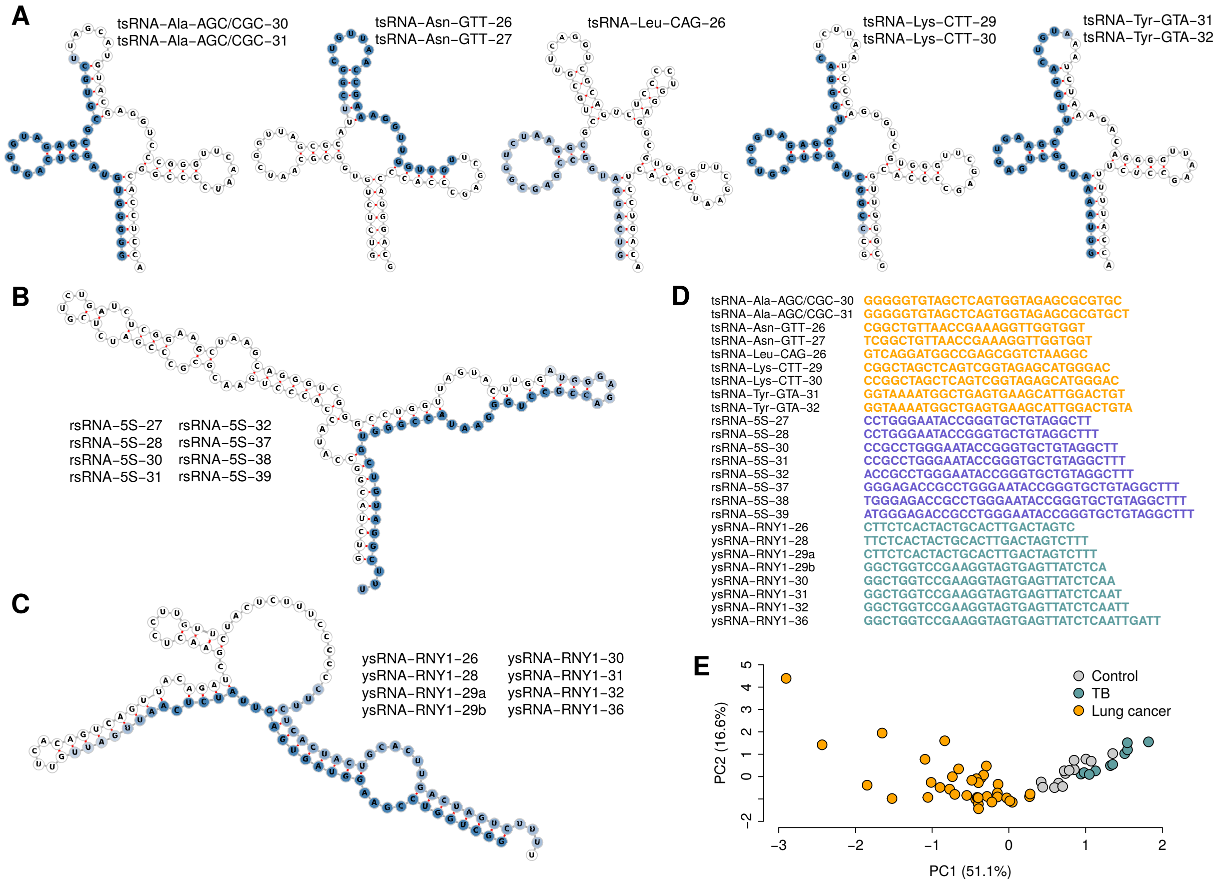

Figure S9. The TRY-RNA signature. (A, B, and C) The tsRNA, rsRNA, and ysRNA species in the signature, respectively. The colored nucleotides indicate the location of the sncRNAs on their corresponding parent RNAs. The darkness of the colors (from light blue to steel blue) indicates the overlap level among different sncRNA species. Nucleotides with higher overlap levels are darkly colored. (D) The sncRNA sequences of the TRY-RNA signature. (E) Principal component analysis of the TRY-RNA signature. PC1: the first principal component; PC2: the second principal component. PC1 significantly differed between the controls and lung cancer patients (t-test: $P < 10^{-10}$), between the controls and TB patients (t-test: $P = 2.1 \times 10^{-4}$), and between the lung cancer and TB patients (t-test: $P < 10^{-10}$).

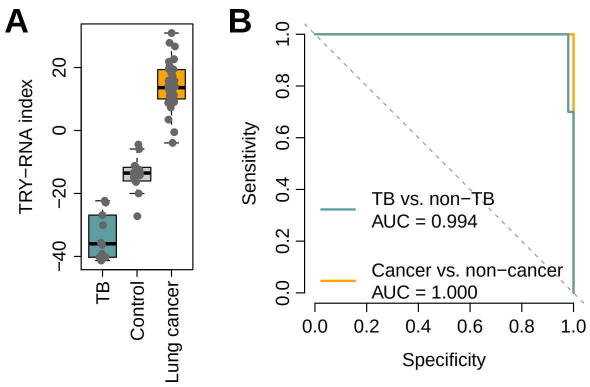

Figure S10. The TRY-RNA index in the discovery cohort. (A) Comparison of the TRY-RNA index among the control, lung cancer, and TB subjects in the discovery cohort. (B) The ROC curve of the TRY-RNA index in distinguishing between lung cancer and non-cancer subjects and between TB and non-TB subjects in the discovery cohort.

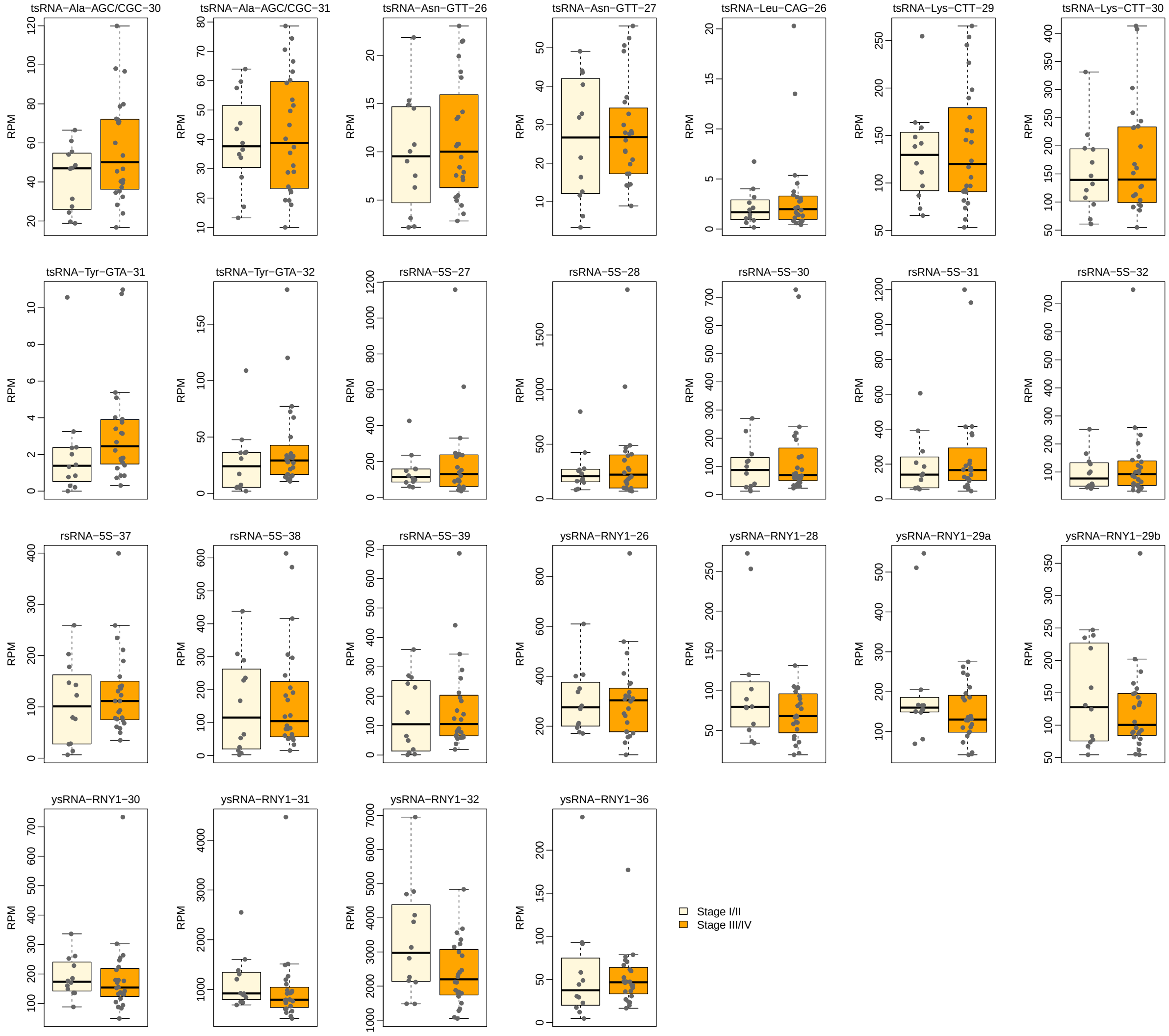

Figure S11. Comparison of the expression of the sncRNA species within the TRY-RNA signature between lung cancer stages. Groupwise comparisons (stage I/II *vs.* III/IV) were performed using a linear model controlling for age and sex. No significant difference was observed (adjusted $P > 0.05$).

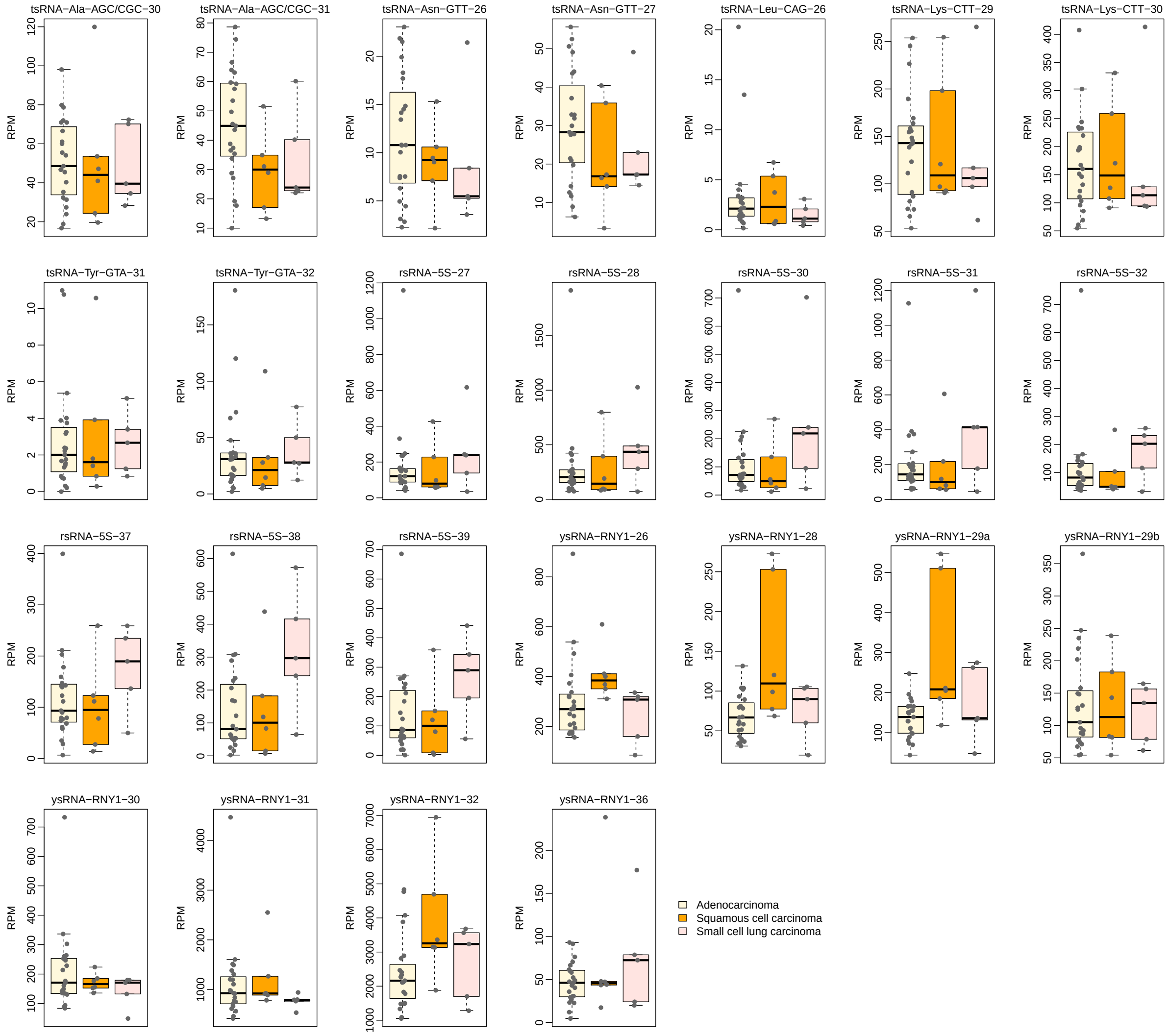

Figure S12. Comparison of the expression of the sncRNA species within the TRY-RNA signature between lung cancer histological types. Groupwise comparisons (*i.e.*, adenocarcinoma *vs.* squamous cell carcinoma, adenocarcinoma *vs.* small cell lung carcinoma, and squamous cell carcinoma *vs.* small cell lung carcinoma) were performed using a linear model controlling for age and sex. Significant difference was only observed for ysRNA-RNY1-28 and ysRNA-RNY1-29a between adenocarcinoma and squamous cell carcinoma (adjusted $P < 0.05$).

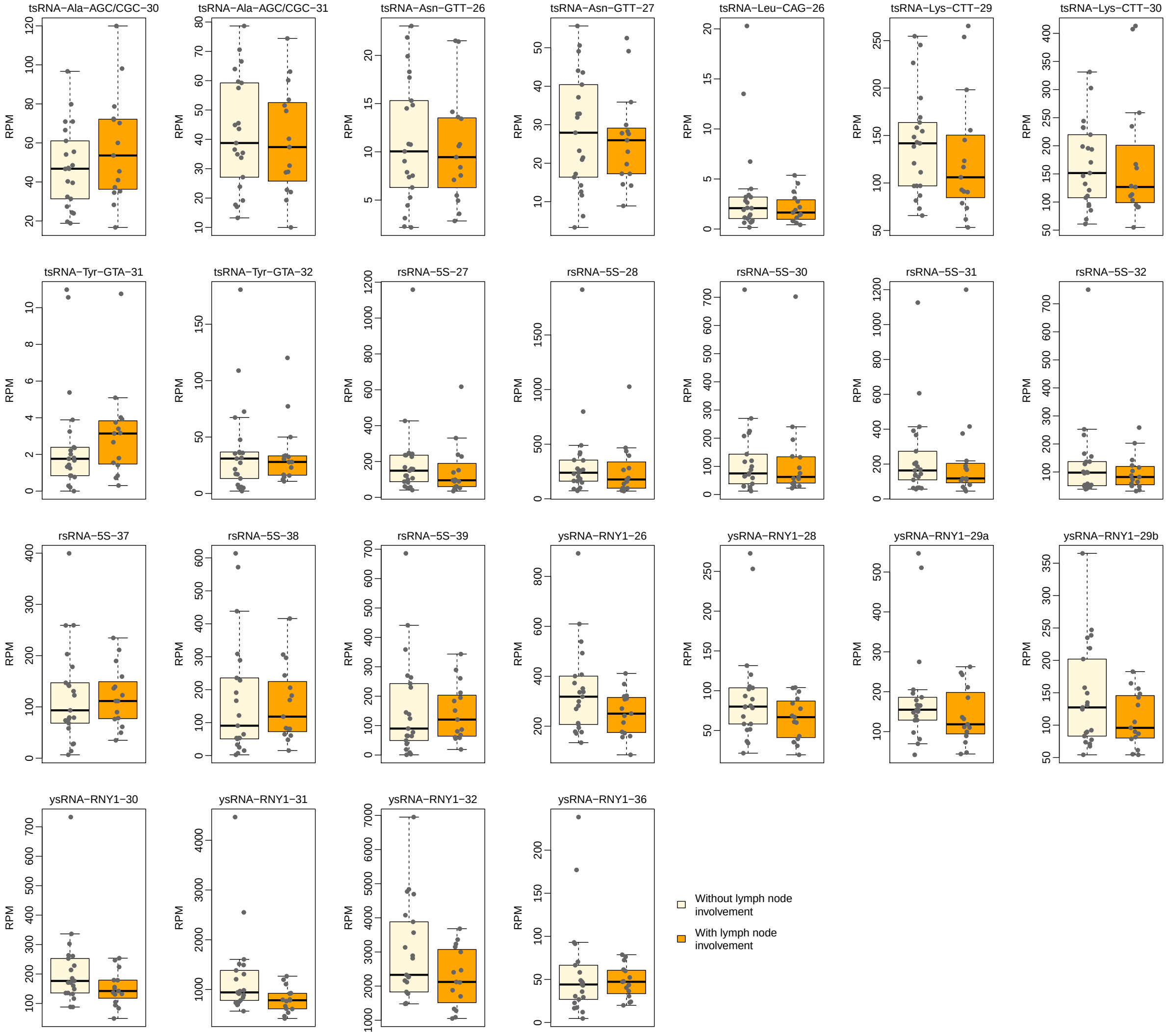

Figure S13. Comparison of the expression of the sncRNA species within the TRY-RNA signature between the lung cancer patients with and without lymph node involvement. Groupwise comparisons were performed using a linear model controlling for age and sex. No significant difference was observed (adjusted $P > 0.05$).

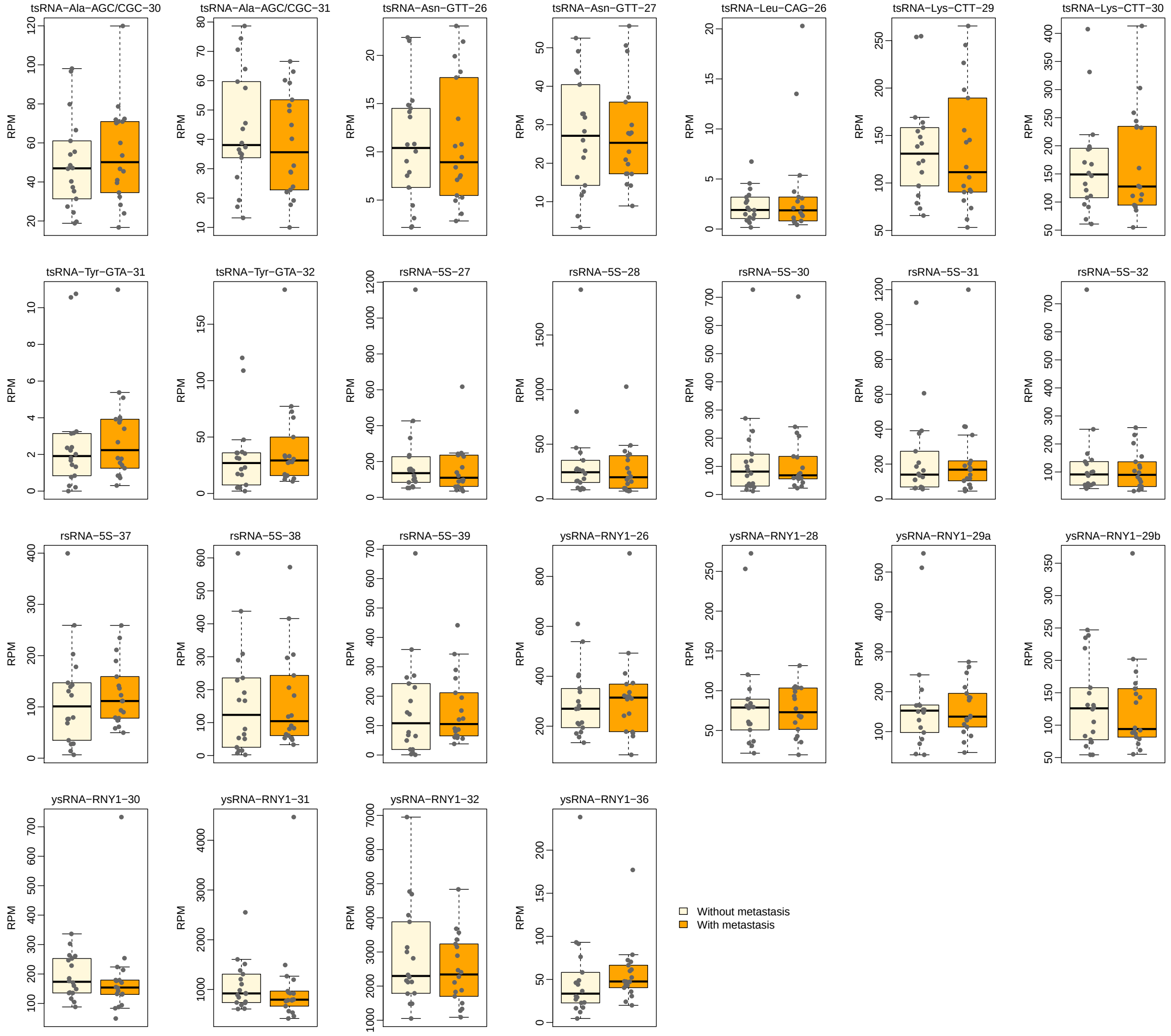

Figure S14. Comparison of the expression of the sncRNA species within the TRY-RNA signature between the lung cancer patients with and without distant metastasis. Groupwise comparisons were performed using a linear model controlling for age and sex. No significant difference was observed (adjusted $P > 0.05$).

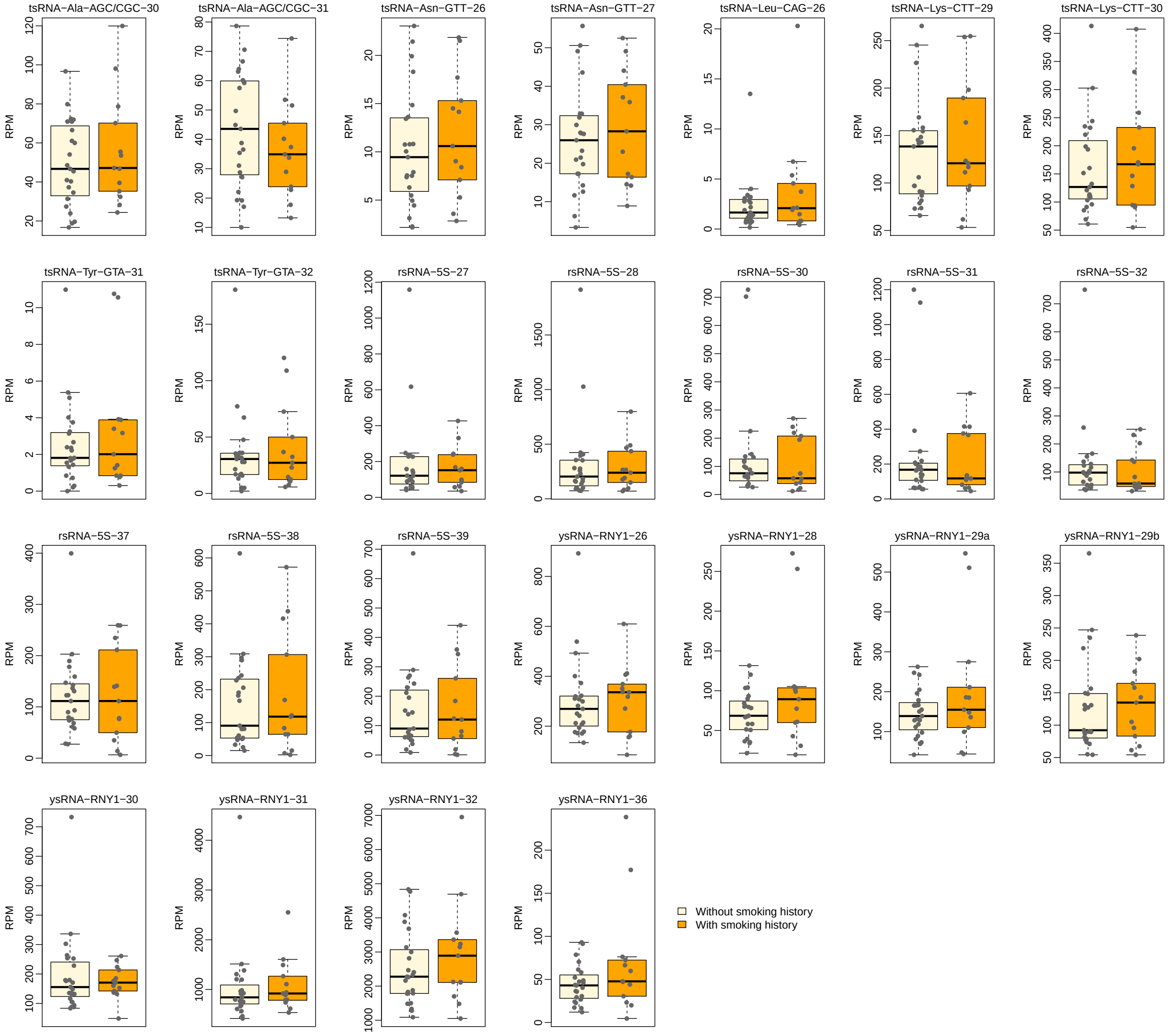

Figure S15. Comparison of the expression of the sncRNA species within the TRY-RNA signature between the lung cancer patients with and without smoking history. Groupwise comparisons were performed using a linear model controlling for age and sex. No significant difference was observed (adjusted $P > 0.05$).

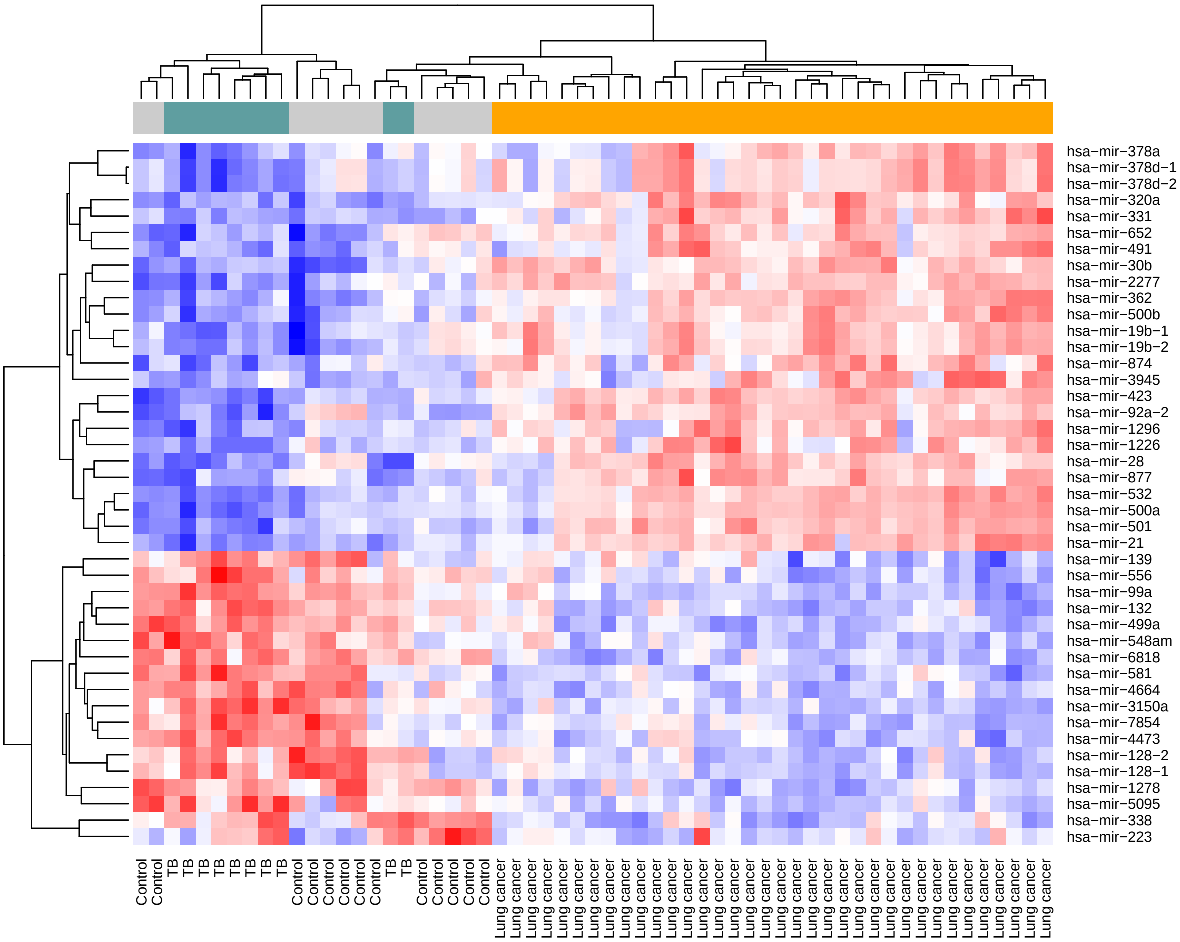

Figure S16. Expression heatmap of the MIR signature in the discovery cohort. Red represents higher expression while blue stands for lower expression.

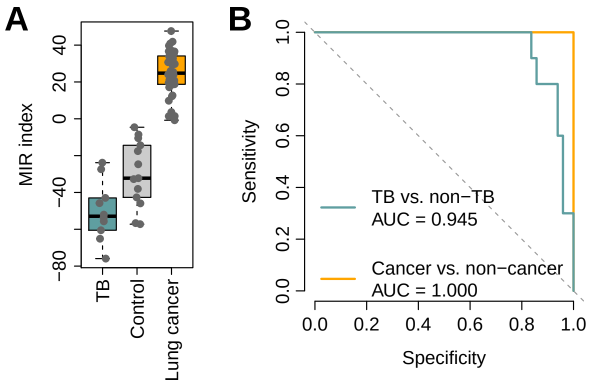

Figure S17. The MIR index in the discovery cohort. (A) Comparison of the MIR index among the control, lung cancer, and TB subjects in the discovery cohort. The MIR index was significantly higher in the lung cancer patients than in the healthy controls (t-test: $P = 2.1 \times 10^{-8}$), while the MIR index of the TB patients was significantly lower than that of the controls (t-test: $P = 8.8 \times 10^{-3}$). (B) The ROC curve of the MIR index in distinguishing between lung cancer and non-cancer subjects and between TB and non-TB subjects in the discovery cohort. The AUC was 1.000 and 0.945 between the cancer and non-cancer subjects and between the TB and non-TB subjects, respectively.

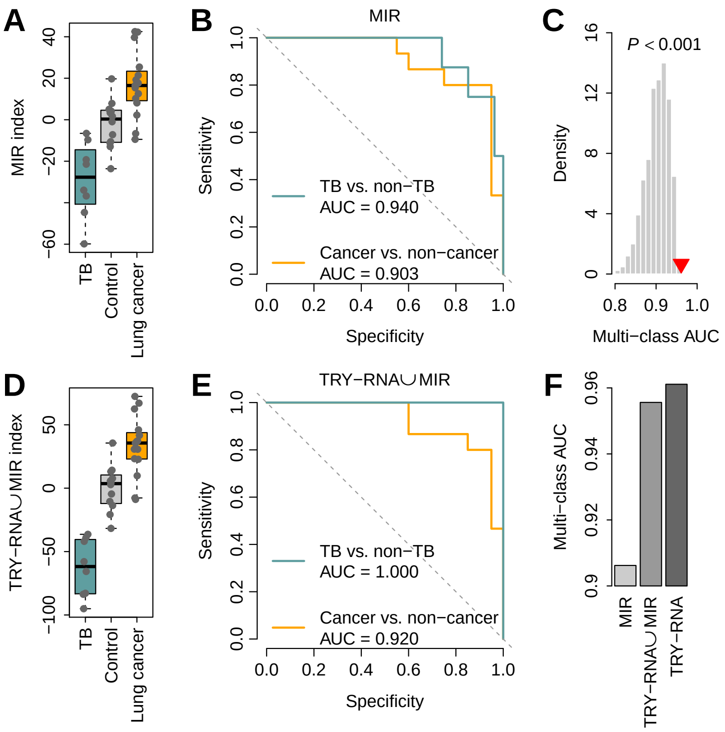

Figure S18. Comparison between the TRY-RNA and MIR signatures. (A) Comparison of the MIR index between the control, lung cancer, and TB subjects in the validation cohort. The MIR index was significantly higher in the lung cancer patients than in the healthy controls (t-test: $P = 1.3 \times 10^{-3}$), while the MIR index of the TB patients was significantly lower than that of the controls (t-test: $P = 3.5 \times 10^{-3}$). (B) The ROC curve of the MIR index in distinguishing between lung cancer and non-cancer subjects and between TB and non-TB subjects in the validation cohort. The AUC was 0.903 between the cancer and non-cancer subjects and 0.940 between the TB and non-TB subjects, suggesting the clinical value of the MIR signature for both pulmonary TB and lung cancer diagnosis, although the AUC was lower than the TRY-RNA signature. (C) The superior classification power of the TRY-RNA signature compared with the MIR signature. The gray histogram shows the distribution of the multi-class AUC values of the 1,000 resampled 25-miRNA signatures randomly picked up from the MIR signature. The red triangle represents the multi-class AUC of the TRY-RNA signature. The right-tailed P-value of the sampling distribution was calculated. As a result, the multi-class AUC of the TRY-RNA signature was significantly larger than that of the random 25-miRNA sets in the validation cohort (right-tailed $P < 0.001$), which suggests a superior classification power of the TRY-RNA signature compared to the MIR signature. (D) Comparison of the TRY-RNA∪MIR index between the control, lung cancer, and TB subjects in the validation cohort. The TRY-RNA∪MIR index was significantly higher in the lung cancer patients than in the healthy controls (t-test: $P = 3.7 \times 10^{-4}$), while the TRY-RNA∪MIR index of the TB patients was significantly lower than that of the controls (t-test: $P = 9.4 \times 10^{-8}$). (E) The ROC curve of the TRY-RNA∪MIR index in distinguishing between lung cancer and non-cancer subjects and between TB and non-TB subjects in the validation cohort. (F) Comparison of the multi-class AUC values between the MIR, TRY-RNA∪MIR, and TRY-RNA signatures in the validation cohort. The performance of the TRY-RNA∪MIR signature was fairly good with AUC of 0.920 and 1.000 between the cancer and non-cancer subjects and between the TB and non-TB subjects, respectively, along with the multi-class $AUC = 0.956$.
