# Supplementary material for: Peripheral blood non-canonical small non-coding RNAs as novel biomarkers in lung cancer
Source: Mol Cancer. 2020 Nov 12;19:159. doi: 10.1186/s12943-020-01280-9 (PMC7659116; doi:10.1186/s12943-020-01280-9)
Supplement: Supplementary file 2 — Additional file 2: Supplementary Tables. Table S1. The human subjects of the discovery cohort. Table S2. The TRY-RNA signature. Table S3. The human subjects of the validation cohort. Table S4. The MIR signature. [file 12943_2020_1280_MOESM2_ESM.pdf]

Table S1. The human subjects of the discovery cohort

ID	Type	Age	Sex	Histology	Stage	Lymph node involvement	Distant metastasis	Smoking history
C_1	Control	59	M					
C_2	Control	53	F					
C_3	Control	80	F					
C_4	Control	59	F					
C_5	Control	50	M					
C_6	Control	68	M					
C_7	Control	41	M					
C_8	Control	34	M					
C_9	Control	48	M					
C_10	Control	54	M					
C_11	Control	56	F					
C_12	Control	53	F					
C_13	Control	31	F					
LC_1	Lung_cancer	62	M	SCLC	4	yes	yes	no
LC_2	Lung_cancer	56	M	ADC	1	no	no	no
LC_3	Lung_cancer	68	F	ADC	3	no	no	no
LC_4	Lung_cancer	69	F	ADC	4	no	yes	no
LC_5	Lung_cancer	46	M	ADC	2	no	no	no
LC_6	Lung_cancer	62	F	ADC	1	no	no	no
LC_7	Lung_cancer	63	F	ADC	3	no	no	no
LC_8	Lung_cancer	42	F	ADC	1	no	no	no
LC_9	Lung_cancer	56	M	ADC	1	no	no	no
LC_10	Lung_cancer	70	M	ADC	3	yes	no	yes
LC_11	Lung_cancer	74	M	ADC	1	no	no	yes
LC_12	Lung_cancer	81	M	ADC	1	no	no	no
LC_13	Lung_cancer	56	M	SCC	1	no	no	yes
LC_14	Lung_cancer	48	F	ADC	4	yes	yes	no
LC_15	Lung_cancer	63	M	SCC	4	yes	yes	yes
LC_16	Lung_cancer	73	M	SCLC	4	no	yes	yes
LC_17	Lung_cancer	66	M	ADC	4	no	yes	no
LC_18	Lung_cancer	73	M	ADC	3	yes	no	yes
LC_19	Lung_cancer	60	M	ADC	4	no	yes	yes
LC_20	Lung_cancer	67	F	Other	3	no	no	no
LC_21	Lung_cancer	67	M	Other	3	yes	no	no
LC_22	Lung_cancer	41	F	ADC	4	yes	yes	no
LC_23	Lung_cancer	68	F	SCLC	4	yes	yes	no
LC_24	Lung_cancer	67	M	ADC	4	yes	yes	yes
LC_25	Lung_cancer	34	F	ADC	4	yes	yes	no
LC_26	Lung_cancer	56	F	ADC	4	no	yes	no
LC_27	Lung_cancer	66	M	SCLC	4	yes	yes	yes
LC_28	Lung_cancer	54	F	SCC	4	yes	yes	no
LC_29	Lung_cancer	72	F	SCC	4	yes	yes	yes

ID	Type	Age	Sex	Histology	Stage	Lymph node involvement	Distant metastasis	Smoking history
LC_30	Lung_cancer	68	M	ADC	4	yes	yes	no
LC_31	Lung_cancer	66	M	ADC	4	no	yes	no
LC_32	Lung_cancer	66	M	SCLC	4	yes	yes	yes
LC_33	Lung_cancer	70	F	ADC	2	no	no	yes
LC_34	Lung_cancer	70	M	SCC	1	no	no	no
LC_35	Lung_cancer	67	M	SCC	1	no	no	yes
LC_36	Lung_cancer	63	F	ADC	1	no	no	no
TB_1	TB	28	M					
TB_2	TB	41	M					
TB_3	TB	48	F					
TB_4	TB	26	M					
TB_5	TB	30	F					
TB_6	TB	30	M					
TB_7	TB	16	F					
TB_8	TB	48	M					
TB_9	TB	55	M					
TB_10	TB	28	M					

Note - ADC: adenocarcinoma; SCC: Squamous cell carcinoma; SCLC: small cell lung carcinoma

Table S2. The TRY-RNA signature

Non-canonical sncRNA	Parent RNA category	Symbol	Weight
GGGGGTGTAGCTCAGTGGTAGAGCGCGTGC	tRNA-Ala	tsRNA-Ala-AGC/CGC-30	1
GGGGGTGTAGCTCAGTGGTAGAGCGCGTGCT	tRNA-Ala	tsRNA-Ala-AGC/CGC-31	1
CGGCTGTTAACCGAAAGGTTGGTGGT	tRNA-Asn	tsRNA-Asn-GTT-26	1
TCGGCTGTTAACCGAAAGGTTGGTGGT	tRNA-Asn	tsRNA-Asn-GTT-27	1
GTCAGGATGGCCGAGCGGTCTAAGGC	tRNA-Leu	tsRNA-Leu-CAG-26	1
CGGCTAGCTCAGTCGGTAGAGCATGGGAC	tRNA-Lys	tsRNA-Lys-CTT-29	1
CCGGCTAGCTCAGTCGGTAGAGCATGGGAC	tRNA-Lys	tsRNA-Lys-CTT-30	1
GGTAAAATGGCTGAGTGAAGCATTGGACTGT	tRNA-Tyr	tsRNA-Tyr-GTA-31	1
GGTAAAATGGCTGAGTGAAGCATTGGACTGTA	tRNA-Tyr	tsRNA-Tyr-GTA-32	1
CCTGGGAATACCGGGTGCTGTAGGCTT	rRNA-5S	rsRNA-5S-27	1
CCTGGGAATACCGGGTGCTGTAGGCTTT	rRNA-5S	rsRNA-5S-28	1
CCGCCTGGGAATACCGGGTGCTGTAGGCTT	rRNA-5S	rsRNA-5S-30	1
CCGCCTGGGAATACCGGGTGCTGTAGGCTTT	rRNA-5S	rsRNA-5S-31	1
ACCGCCTGGGAATACCGGGTGCTGTAGGCTTT	rRNA-5S	rsRNA-5S-32	1
GGGAGACCGCCTGGGAATACCGGGTGCTGTAGGCTTT	rRNA-5S	rsRNA-5S-37	1
TGGGAGACCGCCTGGGAATACCGGGTGCTGTAGGCTTT	rRNA-5S	rsRNA-5S-38	1
ATGGGAGACCGCCTGGGAATACCGGGTGCTGTAGGCTTT	rRNA-5S	rsRNA-5S-39	1
CTTCTCACTACTGCACTTGACTAGTC	YRNA-RNY1	ysRNA-RNY1-26	-1
TTCTCACTACTGCACTTGACTAGTCTTT	YRNA-RNY1	ysRNA-RNY1-28	-1
CTTCTCACTACTGCACTTGACTAGTCTTT	YRNA-RNY1	ysRNA-RNY1-29a	-1
GGCTGGTCCGAAGGTAGTGAGTTATCTCA	YRNA-RNY1	ysRNA-RNY1-29b	-1
GGCTGGTCCGAAGGTAGTGAGTTATCTCAA	YRNA-RNY1	ysRNA-RNY1-30	-1
GGCTGGTCCGAAGGTAGTGAGTTATCTCAAT	YRNA-RNY1	ysRNA-RNY1-31	-1
GGCTGGTCCGAAGGTAGTGAGTTATCTCAATT	YRNA-RNY1	ysRNA-RNY1-32	-1
GGCTGGTCCGAAGGTAGTGAGTTATCTCAATTGATT	YRNA-RNY1	ysRNA-RNY1-36	-1

Table S3. The human subjects of the validation cohort

ID	Type	Age	Sex	Histology	Stage	Lymph node involvement	Distant metastasis	Smoking history
C_validation_1	Control	24	F					
C_validation_2	Control	20	F					
C_validation_3	Control	67	F					
C_validation_4	Control	54	F					
C_validation_5	Control	75	M					
C_validation_6	Control	55	M					
C_validation_7	Control	69	M					
C_validation_8	Control	56	M					
C_validation_9	Control	64	M					
C_validation_10	Control	18	F					
C_validation_11	Control	22	M					
C_validation_12	Control	22	F					
LC_validation_1	Lung_cancer	45	F	ADC	3	no	no	no
LC_validation_2	Lung_cancer	69	M	ADC	2	no	no	yes
LC_validation_3	Lung_cancer	54	F	ADC	1	no	no	no
LC_validation_4	Lung_cancer	50	M	SCC	1	no	no	no
LC_validation_5	Lung_cancer	71	F	ADC	3	no	no	no
LC_validation_6	Lung_cancer	44	M	ADC	4	no	yes	no
LC_validation_7	Lung_cancer	74	M	SCLC	3	yes	no	no
LC_validation_8	Lung_cancer	51	M	ADC	1	no	no	yes
LC_validation_9	Lung_cancer	57	M	ADC	3	yes	no	no
LC_validation_10	Lung_cancer	66	M	SCLC	4	yes	yes	no
LC_validation_11	Lung_cancer	47	F	ADC	2	yes	no	no
LC_validation_12	Lung_cancer	65	M	ADC	4	no	yes	yes
LC_validation_13	Lung_cancer	65	M	SCC	2	no	no	no
LC_validation_14	Lung_cancer	75	F	SCLC	3	yes	no	no
LC_validation_15	Lung_cancer	76	M	SCC	1	no	no	yes
TB_validation_1	TB	18	F					
TB_validation_2	TB	36	M					
TB_validation_3	TB	35	M					
TB_validation_4	TB	74	F					
TB_validation_5	TB	56	M					
TB_validation_6	TB	25	M					
TB_validation_7	TB	23	M					

Note - ADC: adenocarcinoma; SCC: Squamous cell carcinoma; SCLC: small cell lung carcinoma

Table S4. The MIR signature

miRNA	Weight
hsa-mir-1226	1
hsa-mir-1296	1
hsa-mir-19b-1	1
hsa-mir-19b-2	1
hsa-mir-21	1
hsa-mir-2277	1
hsa-mir-28	1
hsa-mir-30b	1
hsa-mir-320a	1
hsa-mir-331	1
hsa-mir-362	1
hsa-mir-378a	1
hsa-mir-378d-1	1
hsa-mir-378d-2	1
hsa-mir-3945	1
hsa-mir-423	1
hsa-mir-491	1
hsa-mir-500a	1
hsa-mir-500b	1
hsa-mir-501	1
hsa-mir-532	1
hsa-mir-652	1
hsa-mir-874	1
hsa-mir-877	1
hsa-mir-92a-2	1
hsa-mir-1278	-1
hsa-mir-128-1	-1
hsa-mir-128-2	-1
hsa-mir-132	-1
hsa-mir-139	-1
hsa-mir-223	-1
hsa-mir-3150a	-1
hsa-mir-338	-1
hsa-mir-4473	-1
hsa-mir-4664	-1
hsa-mir-499a	-1
hsa-mir-5095	-1
hsa-mir-548am	-1
hsa-mir-556	-1
hsa-mir-581	-1
hsa-mir-6818	-1
hsa-mir-7854	-1

miRNA	Weight
hsa-mir-99a	-1
