# Supplementary material for: Peripheral blood non-canonical small non-coding RNAs as novel biomarkers in lung cancer
Source: Mol Cancer. 2020 Nov 12;19:159. doi: 10.1186/s12943-020-01280-9 (PMC7659116; doi:10.1186/s12943-020-01280-9)
Supplement: Supplementary file 3 — Additional file 3. Methods. [file 12943_2020_1280_MOESM3_ESM.pdf]

Methods

Human subjects

This study aimed to develop a non-canonical sncRNA-based molecular signature in human PBMCs differentiating lung cancer patients from healthy controls and pulmonary TB subjects. sncRNA-seq was applied to measure the PBMC ts/rs/ysRNA expression for both the discovery (n = 59) and validation (n = 35) cohorts. All the subjects of this study were of Chinese Han descent. Lung cancer patients were recruited from the First Affiliated Hospital of Bengbu Medical College without receiving adjuvant chemotherapy. Both histological and radiological features were collected for the diagnosis of lung cancer. Active pulmonary TB patients were recruited from the Infectious Disease Hospital of Bengbu City before any TB treatment. The diagnosis of TB was based on established international guidelines [1]. The healthy controls were recruited from the Physical Examination Center of the First Affiliated Hospital of Bengbu Medical College. Subjects with other concurrent infectious diseases were excluded. All subjects were recruited consecutively over time, with the discovery cohort being recruited first followed by the validation cohort. The detailed information is presented in Additional file 1 (Table S1) and Additional file 2 (Table S2). The Ethics Committee of Bengbu Medical College approved this study, with written informed consent obtained from all subjects, which conformed to the standard indicated by the Declaration of Helsinki.

PBMC RNA isolation

We collected PBMCs from the subjects in the discovery and validation cohorts. Five milliliters of anticoagulant peripheral blood was drawn from the ulnar vein of each subject. PBMCs were immediately isolated by the Ficoll-Hypaque density gradient centrifugation method. Briefly,

the blood samples were diluted with RPMI-1640 basic medium at a ratio of 1:1. The diluted blood was added and spread over the Ficoll-Hypaque separation solution at a ratio of 2:1 and then centrifuged at 2,000 revolutions per minute for 20 minutes at room temperature. After centrifugation, the white misty cell layer was collected into a new centrifuge tube and washed twice with RPMI-1640 basic medium at 2,000 revolutions per minute for 5 minutes at 4 ℃. The isolated PBMCs were transferred into 1.5 mL tubes, and 1 mL TRIzol (Ambion, Thermo Fisher Scientific) was added for subsequent total RNA extraction. We extracted total RNA from PBMCs using TRIzol reagent (Invitrogen) and purified it with a mirVana miRNA Isolation Kit (Ambion, Thermo Fisher Scientific) according to the manufacturer's protocol. RNA degradation and contamination were monitored on 1% agarose gels. RNA purity was checked using a NanoPhotometer® spectrophotometer (Implen, CA, USA). RNA concentration was measured using a Qubit® RNA Assay Kit in a Qubit® 2.0 Fluorometer (Life Technologies, CA, USA). RNA integrity was assessed by the RNA Nano 6000 Assay Kit of the Agilent Bioanalyzer 2100 system (Agilent Technologies, CA, USA). Only the RNA samples with RNA integrity values > 6 were retained for further study.

sncRNA-seq library preparation

A total of ~2 μg total RNA per sample was used as the input for the sncRNA-seq libraries. The sncRNA-seq libraries were constructed using the NEBNext® Multiplex Small RNA Library Prep Set for Illumina® (NEB, USA). The NEB 3' SR adaptor was ligated to the 3' end of sncRNAs, followed by the SR RT Primer being hybridized to the excess 3' SR adaptor, which transformed the single-stranded DNA adaptor into double-stranded DNA. The 5' end adapter was ligated to the 5' ends of sncRNAs, followed by the first cDNA strand being synthesized using M-MuLV Reverse

Transcriptase. PCR amplification was performed using LongAmp Taq 2X Master Mix for 11-13 cycles, and the products were purified on an 8% polyacrylamide gel (100 V, 80 minutes). DNA fragments were recovered and dissolved in 8 μL elution buffer. The qualified libraries, which were assessed by an Agilent Bioanalyzer 2100, were amplified on the cBot to generate the cluster on the flow cell. The amplified flow cell was sequenced (single-end) on the Illumina System with a read length of 50 nucleotides (nts).

sncRNA-seq data processing

Our newly developed pipeline, *SPORTS1.0* (https://github.com/junchaoshi/sports1.0) [2], was used to parse the raw sncRNA-seq data. *SPORTS1.0* was designed to optimize the annotation and quantification of non-canonical small RNAs from sncRNA-seq data. Briefly, *SPORTS1.0* was used to output clean reads by removing sequence adapters and discarding sequences with lengths beyond the defined range and those with bases other than ATUCG. The clean reads were sequentially mapped against miRbase [3], the rRNA/YRNA database (obtained from NCBI), and GtRNAdb [4]. Because miRNAs were dominant among the sequencing reads, we summarized the reads per million (*RPM*) values for the sncRNA species with lengths ≤ 25 nts and > 25 nts separately. The miRNA-based signature was developed from sequencing reads with lengths ≤ 25 nts, while the signature composed of ts/rs/ysRNAs was derived from sequencing reads with lengths > 25 nts. We only retained the non-canonical sncRNAs with at least one read in at least 10 samples. We further grouped non-canonical sncRNA species, *i.e.*, ts/rs/ysRNAs, into individual subcategories according to the parent large RNAs from which they originated. The sncRNA subcategories with fold change < 2 between the control and lung cancer groups were excluded from further analyses. We used a linear model controlling for age and sex [5] to compare the

expression of each sncRNA subcategory between the control and lung cancer groups. The *Benjamini-Hochberg* procedure was used for *P*-value correction. The same linear model controlling for age and sex was also used to compare the expression of each sncRNA subcategory between the pulmonary TB and lung cancer groups. To identify the sncRNA species that were differentially expressed between the controls and lung cancer patients and between the controls and TB patients, we employed the *edgeR* tool [6] controlling for age and sex. The sncRNA species (mean *RPM* > 1) with a false discovery rate < 0.01 were deemed differentially expressed.

Developing the molecular signatures

To develop the TRY-RNA signature, only the sncRNA species differentially expressed between the controls and lung cancer patients and between the pulmonary TB and lung cancer patients were retained. To avoid potential biases caused by RNA size fractionation procedures, we further excluded sncRNA species with lengths ≥ 40 nts. For each tsRNA subcategory, we only collected the top two tsRNA species with the highest average expression levels across all the PBMC samples if there was more than one tsRNA species within this subcategory. In total, nine tsRNA species were prioritized: tsRNA-Ala-AGC/CGC-30 and tsRNA-Ala-AGC/CGC-31 belonging to tsRNA-Ala, tsRNA-Asn-GTT-26 and tsRNA-Asn-GTT-27 belonging to tsRNA-Asn, tsRNA-Leu-CAG-26 belonging to tsRNA-Leu, tsRNA-Lys-CTT-29 and tsRNA-Lys-CTT-30 belonging to tsRNA-Lys, and tsRNA-Tyr-GTA-31 and tsRNA-Tyr-GTA-32 belonging to tsRNA-Tyr. For rsRNA-5S and ysRNA-RNY1, we collected the RNA species with mean *RPM* > 50, yielding eight rsRNA species, rsRNA-5S-27, rsRNA-5S-28, rsRNA-5S-30, rsRNA-5S-31, rsRNA-5S-32, rsRNA-5S-37, rsRNA-5S-38, and rsRNA-5S-39, and eight ysRNA species, ysRNA-RNY1-26, ysRNA-RNY1-28, ysRNA-RNY1-29a, ysRNA-RNY1-29b, ysRNA-RNY1-

30, ysRNA-RNY1-31, ysRNA-RNY1-32, and ysRNA-RNY1-36. We also examined the expression profile of miRNAs among the control, lung cancer, and pulmonary TB patients in the discovery cohort. In total, 43 miRNA species were found to be differentially expressed between the controls and lung cancer patients and between the TB and lung cancer patients. We designated these 43 miRNAs as the MIR signature.

The TRY-RNA, MIR, and TRY-RNA∪MIR indices

We applied a scoring scheme used in our previous studies to assign each human subject a TRY-RNA index [7, 8]:

$$I_{TRY-RNA} = \sum_{i=1}^{25} w_i(e_i - \mu_i)/\tau_i$$

Here, $I_{TRY-RNA}$ was the TRY-RNA index; w_i was the weight of non-canonical sncRNA *i* within the TRY-RNA signature, as shown in Supplementary Table S2, which was derived from the discovery cohort (w_i = 1 if sncRNA *i* was upregulated in the lung cancer patients relative to the controls, while w_i = -1 if sncRNA *i* was downregulated in the lung cancer patients); e_i denoted the expression level of sncRNA *i*; and μ_i and τ_i were the mean and standard deviation of the expression of sncRNA *i* across all the samples, respectively. A higher TRY-RNA index implies a higher likelihood of lung cancer. Similarly, the MIR index was defined as:

$$I_{MIR} = \sum_{i=1}^{43} w_i(e_i - \mu_i)/\tau_i$$

Here, I_{MIR} was the MIR index; w_i was the weight of miRNA *i* within the MIR signature as shown in Supplementary Table S4, which was also derived from the discovery cohort (w_i = 1 if miRNA *i* was upregulated in the lung cancer patients relative to the controls, while w_i = -1 if miRNA *i* was

downregulated in the lung cancer patients); e_i denoted the expression level of miRNA *i*; and μ_i and τ_i were the mean and standard deviation of the expression of miRNA *i* across all the samples, respectively. Finally, the TRY-RNA∪MIR index was defined as:

$$I_{TRY-RNA\cup MIR} = \sum_{i=1}^{68} w_i(e_i - \mu_i)/\tau_i$$

Here, $I_{TRY\text{-}RNA\cup MIR}$ was the TRY-RNA∪MIR index; w_i was the weight of RNA *i* within the TRY-RNA∪MIR signature (including 25 ts/rs/ysRNA and 43 miRNA species); e_i denoted the expression level of RNA *i*; and μ_i and τ_i were the mean and standard deviation of the expression of RNA *i* across all the samples, respectively.

Resampling test

Because the size between the TRY-RNA and MIR signatures was different (25 ts/rs/ysRNA species *vs*. 43 miRNA species), to perform a fair comparison between the two signatures, we conducted a resampling test by randomly selecting 25 miRNA sequences from the MIR signature 1,000 times. For each random 25-miRNA signature, we recalculated the MIR index for each subject, and a multi-class *AUC* was computed among the control, lung cancer, and TB groups according to the generalization model proposed by Hand and Till [9], which represented the classification power of the random signature.

Statistical analysis

All statistical analyses were performed using the *R* platform. Correlations between continuous variables were measured by *Spearman*'s rank correlation test using the "cor.test" function. Student's *t*-test was performed for groupwise comparisons of normal distributions, using

the “t.test” function. A linear model controlling for age and sex was applied to prioritize the differentially expressed non-canonical sncRNA subcategories using the “lm” function. If multiple testing should be accounted for, the *Benjamini-Hochberg* procedure was applied for *P*-value correction using the “p.adjust” function. Principal component analysis on the expression data of the TRY-RNA signature was performed using the “dudi.pca” function within the package “ade4”. The *AUC* and multi-class *AUC* values were computed using the “roc” and “multiclass.roc” functions respectively, within the package “pROC”.

References for Methods

1. Lewinsohn DM, Leonard MK, LoBue PA, Cohn DL, Daley CL, Desmond E, Keane J, Lewinsohn DA, Loeffler AM, Mazurek GH, et al: **Official American Thoracic Society/Infectious Diseases Society of America/Centers for Disease Control and Prevention Clinical Practice Guidelines: Diagnosis of Tuberculosis in Adults and Children.** *Clin Infect Dis* 2017, **64:**111-115.
2. Shi J, Ko EA, Sanders KM, Chen Q, Zhou T: **SPORTS1.0: A Tool for Annotating and Profiling Non-coding RNAs Optimized for rRNA- and tRNA-derived Small RNAs.** *Genomics Proteomics Bioinformatics* 2018, **16:**144-151.
3. Kozomara A, Griffiths-Jones S: **miRBase: annotating high confidence microRNAs using deep sequencing data.** *Nucleic Acids Res* 2014, **42:**D68-73.
4. Chan PP, Lowe TM: **GtRNAdb 2.0: an expanded database of transfer RNA genes identified in complete and draft genomes.** *Nucleic Acids Res* 2016, **44:**D184-189.

5. McDonough JE, Kaminski N, Thienpont B, Hogg JC, Vanaudenaerde BM, Wuyts WA: **Gene correlation network analysis to identify regulatory factors in idiopathic pulmonary fibrosis.** *Thorax* 2019, **74:**132-140.

6. Robinson MD, McCarthy DJ, Smyth GK: **edgeR: a Bioconductor package for differential expression analysis of digital gene expression data.** *Bioinformatics* 2010, **26:**139-140.

7. Qian Z, Liu H, Li M, Shi J, Li N, Zhang Y, Zhang X, Lv J, Xie X, Bai Y, et al: **Potential Diagnostic Power of Blood Circular RNA Expression in Active Pulmonary Tuberculosis.** *EBioMedicine* 2018, **27:**18-26.

8. Qian Z, Lv J, Kelly GT, Wang H, Zhang X, Gu W, Yin X, Wang T, Zhou T: **Expression of nuclear factor, erythroid 2-like 2-mediated genes differentiates tuberculosis.** *Tuberculosis (Edinb)* 2016, **99:**56-62.

9. Hand DJT, R.J.: **A Simple Generalisation of the Area Under the ROC Curve for Multiple Class Classification Problems.** *Mach Learn* 2001, **45:**171-186.
